# A Bi-level optimization method for integrated warehouse design, storage location assignment, and order picking

**DOI:** 10.1016/j.mex.2025.103595

**Published:** 2025-08-28

**Authors:** Mohammadreza Farhadi Moghadam, Kaveh Khalili damghani, Vahidreza Ghezavati, Alireza Rashidi Komijan

**Affiliations:** Department of Industrial Engineering, ST.C., Islamic Azad University, Tehran, Iran

**Keywords:** Warehouse design, Storage location assignment problem, Order picking, Bi level optimization

## Abstract

This study proposes a novel bi-level optimization model for integrated warehouse planning, encompassing shelving technology selection, storage location assignment, and order picking decisions. Unlike previous studies that have largely treated these components in isolation, this research addresses the interdependencies across strategic, tactical, and operational planning horizons in a unified framework. The proposed model captures the complex interaction between layout design and real-time order fulfillment processes, ensuring a holistic optimization of warehouse performance. At the upper (leader) level, the model determines the optimal allocation of available space to three shelving technologies—wide-aisle pallet racks, narrow-aisle pallet racks, and mobile racks—based on construction costs and space utilization efficiency. These technologies differ significantly in setup costs, equipment requirements, and spatial constraints. The goal of the leader model is to minimize construction costs while meeting shelf capacity needs driven by fluctuating input/output volumes. At the lower (follower) level, the model integrates item-to-location assignment and order picking path optimization, taking into account probabilistic inbound and outbound flows over multiple periods. Product allocation to storage positions and transportation equipment is optimized with respect to equipment capacities and inventory dynamics. Additionally, the follower model includes constraints for order balancing, product placement and retrieval, vehicle loading, and sub-tour elimination in picking paths. The required shelf capacity, initially assumed as an input, is iteratively updated based on the follower’s output and reintroduced to the leader level, enabling a feedback-driven optimization loop. To solve this hierarchical problem structure, an enumerative heuristic method is adopted. The leader’s solution space is discretized, and for each candidate solution, the follower model is solved using GAMS. This approach enables the identification of near-optimal configurations with manageable computational effort while maintaining solution feasibility and interpretability.

The innovations of this study are threefold: (1) it is among the first to integrate all three critical components of warehouse planning—shelving technology, storage location assignment, and order picking—in a bi-level structure; (2) it incorporates probabilistic modeling to account for demand uncertainty in each planning period; and (3) it proposes a practical solution methodology that balances cost, performance, and computational tractability.

Numerical results show that the integrated model significantly outperforms traditional sequential approaches by reducing construction and transportation costs and improving space utilization. Sensitivity analyses confirm the robustness of the model in response to variations in demand patterns and transportation costs. The proposed framework offers warehouse designers and decision-makers a practical and data-driven tool for optimizing warehouse layout and operations under uncertainty.

## Graphical abstract

**Figure:**
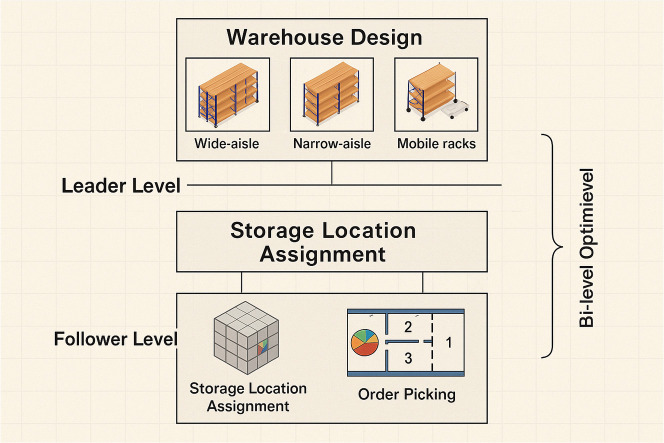

Specifications table

**Table:** 

**Subject area**	Engineering
**More specific subject area**	*Warehouse design, Storage assignment, Order picking*
**Name of your method**	*Bi-level optimization with enumeration-based heuristic*
**Name and reference of original method**	*Numbered*
**Resource availability**	*GAMS model available upon request*

## Background

Warehouse operations encompass three closely related components: warehouse design and shelving technology selection, storage location assignment, and order picking. While each of these has been extensively explored in the literature, they are often studied in isolation, which limits the potential for achieving globally optimal solutions. The foundational work by Hausman et al. [1] emphasized the growing strategic importance of warehouse systems, transitioning the focus from productivity to cost efficiency. Since then, numerous models have addressed specific operational aspects of warehouses.

For example, Öztürk [2] developed a bi-level model that integrated storage location assignment with order picking, highlighting the effectiveness of genetic algorithms for real-time optimization. Similarly, Yang et al. [3] focused on joint scheduling and storage decisions in automated storage and retrieval systems, validating the benefits of integrated approaches. Dijkstra and Roodbergen [4] proposed a dynamic programming model that jointly considered storage assignment and routing strategies, while Van Gils et al. [5] reinforced the importance of synchronizing tactical and operational decisions in high-volume picking systems.

Several more recent studies, such as those by Yener and Yazgan [6] and Zheng et al. [7], incorporated customer demand patterns and item co-occurrence data into order picking models to reduce travel time. Kobler et al. [8] attempted to bridge the integration gap by introducing an iterative framework that considered layout, storage, and picking simultaneously. Habibi-Tostani et al. [9] addressed a bi-level structure incorporating storage policies and crane scheduling, while Gong Zhang et al. [10] and Mojaver Tabrizi et al. [11] acknowledged the computational challenges of full integration, suggesting heuristic approaches as practical alternatives.

More recently, with the rise of robotic and data-driven fulfilment systems, studies like those of Xuan et al. [12] and Ding et al. [13] have demonstrated the effectiveness of using historical data and dynamic rack positioning to optimize item placement and reduce congestion. However, these contributions still largely concentrate on operational-level decisions and fail to capture the broader strategic implications of warehouse design.

Despite these advancements, a comprehensive modelling approach that simultaneously integrates all three levels—warehouse design and shelving technology selection (strategic), storage location assignment (tactical), and order picking (operational)—is missing in the literature. The absence of such a framework overlooks the critical interdependencies among these decisions. For example, shelving technology directly affects item accessibility and picker routes, yet this linkage is rarely considered in integrated models.

Therefore, a significant research opportunity exists to develop a unified bi-level optimization model that combines these three components. Such an integrated approach would reflect real-world decision hierarchies, enhance system-wide efficiency, and provide practical guidance for warehouse planners and managers aiming to reduce costs and improve service levels.

## Method details

Warehouses and distribution centres constitute vital components of modern supply chain and logistics networks. Core operations in these facilities include goods receiving, inventory management, storage location assignment, order picking, sorting, packaging, and shipment. These functions are essential for maintaining accuracy, speed, and cost-efficiency across the supply chain, directly impacting service levels and customer satisfaction. Recent developments—such as globalization, heightened customer expectations, rising labour costs, and limited availability of industrial land—have intensified the demand for more efficient warehouse operations, particularly in the order picking process. Order picking, defined as the retrieval of items from storage locations to fulfil customer orders, is recognized as one of the most labour-intensive and cost-driving activities within warehouse environments. Order picking systems are generally classified into two categories: picker-to-part systems, where the worker travels to the item location, and part-to-picker systems, in which items are brought to the operator via automation. Despite growing access to automation technologies, picker-to-part systems remain predominant in practice, primarily due to their operational flexibility and lower initial investment requirements. As a result, a significant portion of warehouses continues to rely on manual labour for order picking tasks, emphasizing the need for practical and scalable optimization approaches.

This study adopts a picker-to-part system, wherein the operator moves through the warehouse to retrieve items required for fulfilling customer orders. Order picking is widely acknowledged as one of the most time-consuming and cost-intensive operations within warehouse environments. From an optimization perspective, the order picker routing problem is generally modelled as a variant of the classical Traveling Salesman Problem without capacity constraints. To enhance the efficiency of the order picking process—which plays a pivotal role in overall warehouse productivity—numerous mathematical models and innovative optimization techniques have been proposed in the literature. A fundamental approach to improving order picking performance is the storage location assignment, which determines how items are allocated to specific locations within the warehouse. The Storage Location Assignment Problem is categorized as an NP-hard problem and is often addressed using heuristic or metaheuristic algorithms due to its computational complexity. In practice, storage location assignment serves as a critical input to the order picking process. While storage location assignment is generally classified as a tactical-level planning problem, order picking is considered an operational-level planning issue. The interdependence between these two decision-making levels underscores the importance of integrated modelling approaches for achieving superior warehouse performance.

The arrangement of items and their storage location assignment are closely linked to the type of shelving technology adopted in the warehouse. Shelving technology and overall warehouse design are considered strategic decisions with a long-term planning horizon. Modifications at this level are typically capital-intensive and difficult to implement once established. Key design elements—such as equipment selection, building structure, layout policies, and space utilization efficiency—are inherently influenced by the chosen shelving technology. Appropriate selection of shelving technology can significantly reduce transportation costs during the order picking process. In conventional warehouse layouts, wide aisles are commonly employed to accommodate general-purpose material handling equipment. Although such configurations involve relatively low setup costs, they are associated with low space utilization efficiency. Conversely, narrow aisle designs improve space utilization but require more specialized equipment, thereby increasing setup costs. An alternative shelving technology involves the use of mobile racking systems, where shelving units are mounted on movable bases, eliminating the need for fixed aisles. While this approach offers maximum space efficiency, it involves high capital costs and typically limits the height of the storage units, thus requiring less specialized equipment compared to narrow aisle systems. Warehouse planning is generally structured across three planning horizons: long-term, medium-term, and short-term. As outlined, warehouse design and shelving technology selection fall under long-term strategic planning. The storage location assignment problem is categorized as a medium-term tactical issue, while order picking is considered a short-term operational activity. Despite their different planning horizons, these components are highly interdependent, and isolated analysis of each may lead to suboptimal system performance.

To address this limitation, the present study proposes an integrated modelling framework that simultaneously considers warehouse design, shelving technology selection, storage location assignment, and order picking. By acknowledging the interdependencies among these decisions, the model aims to enhance overall system efficiency. Notably, a comprehensive approach that concurrently integrates these elements has not been adequately addressed in the existing literature.

Numerous studies have demonstrated that addressing warehouse design, storage location assignment, and order picking in isolation leads to suboptimal outcomes in terms of overall system performance. In particular, physical warehouse design has a direct influence on storage location decisions and the structure of order picking routes. Therefore, adopting an integrated and simultaneous approach to these interrelated decisions can substantially reduce total travel distances, shorten order fulfilment times, and enhance the efficiency of both human resources and material handling equipment. In this context, Van Gils et al. [5] conducted a comprehensive review and classification of the literature on the integration of tactical planning and order picking operations in picker-to-item systems. Their objective was to explore the interdependencies among planning decisions, with findings indicating that most prior studies focused primarily on the combination of zoning and routing problems. Earlier, Caron et al. [14] investigated the combined problem of order picker routing and storage location assignment, highlighting the benefits of joint decision-making in improving warehouse performance. Strategic planning decisions—such as warehouse layout and equipment configuration—have cascading effects on tactical and operational planning issues. Accordingly, reviewing studies that examine the impact of strategic-level decisions on medium- and short-term operations can provide valuable insights for designing more efficient and responsive warehouses. Boysen et al. [15] further illustrated that when warehouse layout is determined without considering order picking paths or storage assignment strategies, the resulting movement of operators and transport equipment is far from optimal. This misalignment leads to increased picking time, energy consumption, and labour costs. Similarly, Roodbergen et al. [16] observed that failure to account for actual order patterns and demand variability during layout design can cause certain warehouse zones to become congested while others remain underutilized, thereby decreasing space efficiency and creating operational bottlenecks. In sum, the lack of integration among warehouse design, storage location assignment, and order picking decisions not only increases operational costs and reduces efficiency but also hinders the fulfilment of broader supply chain strategic objectives, particularly in competitive and dynamic market environments.

**Fig. 1:**
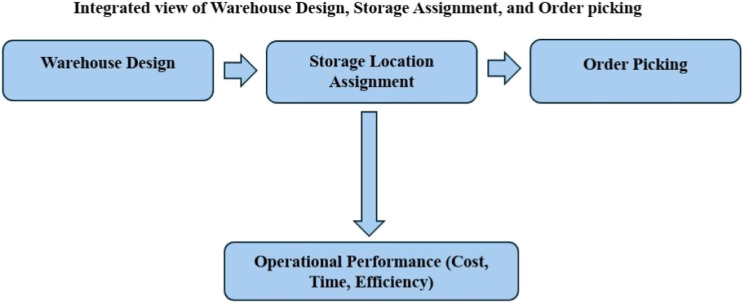
Conceptual Map of the Integrated Relationship among the Three Key Components of Warehousing.

Fig. 1 presents a conceptual framework illustrating the integrated relationship among the three fundamental components of warehouse operations: warehouse design, storage location assignment, and order picking. Warehouse design encompasses decisions regarding the physical configuration of the facility, including the positioning of aisles, inbound and outbound points, and the overall spatial layout. These design choices have a direct impact on the internal structure and feasibility of efficient space utilization. Based on this physical configuration, storage location assignment determines the optimal placement of items within the warehouse to facilitate efficient retrieval. Subsequently, the order picking process—which involves retrieving items to fulfil customer orders—is highly dependent on both the storage layout and the spatial arrangement established during the design phase. This process significantly influences critical operational outcomes such as response time, labour costs, and customer satisfaction. An integrated and optimized approach to these three interrelated components not only enhances overall warehouse performance but also results in cost reduction, time savings, and greater operational efficiency across the warehouse system.

In Table 1, the key factors influencing each of the three main components in the integrated warehousing model are presented.

**Table 1:** Key factors influencing the main components of the integrated warehousing model.

Impact on Overall Performance	Key Influencing Factors	System Component
Providing the physical basis for downstream decision-making, reducing pathway interference	Type of goods, warehouse dimensions, number of aisles, entry and exit locations, type of equipment, shelving technology type	Warehouse Design
Direct impact on picking time, energy consumption, and space optimization	Goods classification, inventory turnover rate, available space	Storage location assignment
Reducing order picking time, enhancing human productivity, improving service level	Number of orders, accessible routes, type of picking system	Order Picking

Table 2 presents the structured representation of the mutual interactions among the three core components of warehousing. This table illustrates how warehouse design influences the structure of storage location assignment and order picking, how storage location assignment shapes the order picking and time, and how order picking provides feedback for optimizing warehouse design and layout.

**Table 2:** Mutual interactions among the three core components of warehousing.

	Warehouse Design	Storage Location Assignment	Warehouse Design
Warehouse Design	—	Limited impact due to capacity and layout constraints	Feedback for layout optimization
Storage Location Assignment	Defining the zones for storage location assignment	—	Suggesting reallocation of high-frequency items
Order Picking	Designing the structure of picking routes	Direct influence on sequencing and picking time	—

In previous research, deterministic models have been successfully applied to a variety of warehouse-related problems. However, there remains a misconception that stochastic approaches are confined to traditional probabilistic frameworks. In a notable review, De Koster [17] examined probabilistic modelling techniques in warehouse operations, highlighting the importance of accounting for demand fluctuations and treating inbound and outbound movement rates as probabilistic variables. The present study employs a bi-level optimization structure, where the follower-level problem—comprising storage location assignment and order picking—is nested within the leader-level problem, which involves decisions related to warehouse design and shelving technology selection. To evaluate both computational performance and overall system efficiency, multiple combinations of order quantities, cost structures, and transportation equipment were tested. The primary objective at the leader level is to determine the optimal warehouse configuration, particularly the selection of shelving technology. This study considers three widely used shelving systems: wide aisle racks, narrow aisle racks, and mobile racks. Each of these technologies offers distinct characteristics in terms of space utilization, required equipment, and associated construction costs. Generally, higher space utilization efficiency is achieved at the expense of increased setup costs and more specialized equipment. Given the increasing scarcity and cost of industrial land, the optimal shelving strategy—or a combination of technologies—is selected to maximize space efficiency while minimizing long-term operational costs. Shelving technology has a direct influence on how items are stored, which subsequently affects internal material handling costs and the performance of the order picking process. Therefore, adopting an integrated optimization approach is essential to capture the interdependencies between warehouse design, storage location assignment, and order picking. This holistic view ensures that all relevant costs and operational constraints are considered simultaneously. In contrast, treating these decisions in isolation can lead to suboptimal outcomes, where hidden costs remain unaddressed, and global system efficiency is compromised.

Given the multi-level structure of the problem and the involvement of various planning horizons, a bi-level optimization approach is employed to address the model. Bi-level optimization is a hierarchical framework in which the overall problem is structured into two interconnected levels, each with its own objective function and decision variables. Typically, the leader level initiates the decision-making process, while the follower level optimizes its decisions in response to the leader's choices.

In real-world warehouse operations, strategic and operational decisions are often made at distinct managerial levels. Bi-level optimization effectively captures this hierarchical decision-making process, enabling more accurate modelling of practical scenarios. The leader level—typically represents long-term strategic or tactical decisions—considers the anticipated response of the follower level, which is responsible for short-term operational decisions. This structure promotes coordination and alignment across different planning levels and enhances system-wide decision quality. By evaluating how follower-level outcomes respond to leader-level decisions, warehouse managers can gain deeper insights into the sensitivity and robustness of their strategic plans. This allows for the development of more flexible and resilient systems, particularly in the presence of operational uncertainties and dynamic environments. Moreover, the explicit interaction between levels encourages collaborative decision-making, where each level can recognize and accommodate the objectives of the other. Because the leader anticipates the reaction of the follower in advance, the risk of suboptimal or infeasible decisions is reduced. Consequently, the solutions generated by bi-level optimization are typically more implementable and better aligned with real-world operational constraints. In this study, a heuristic enumerative method is adopted to solve the bi-level problem. This approach is selected due to its simplicity, ability to find globally optimal solutions within discrete decision spaces, and its compatibility with metaheuristic techniques applied at the follower level. Unlike methods that require reformulating the problem into a single-level equivalent, the enumerative approach retains the hierarchical structure, making it more intuitive and easier to implement. The enumerative method involves a comprehensive and systematic exploration of the leader-level decision variable space. For each candidate solution at the leader level, the corresponding follower-level problem is solved independently. Ultimately, the leader-follower pair that yields the most favourable value of the upper-level objective function is selected as the optimal solution. This process, while computationally intensive for large-scale problems, ensures high accuracy and preserves the integrity of the bi-level model structure.

The main innovation of this study lies in the development of an integrated model that combines shelving technology selection with storage location assignment and order picking. Unlike previous research that typically treats these decisions independently, this study addresses them simultaneously within a unified framework. The proposed problem is formulated as a multi-period model that incorporates three distinct planning horizons: long-term (shelving technology selection and warehouse design), mid-term (storage location assignment), and short-term (order picking operations). Furthermore, to reflect real-world conditions more accurately, the model incorporates uncertainty in the inbound and outbound flow of items, treating this parameter as probabilistic. This approach enhances the model's applicability in dynamic warehouse environments and supports more robust and data-driven decision-making.

The Bi level optimization is presented parametrically as follows. The mechanism for solving a bi-level optimization model can be written as:

$$Min_{x\epsilon X}\;F\left(x,y^{*}\left(x,\theta \right),\theta \right)$$

$$s.b\;G\left(x,y^{*}\left(x,\theta \right),\theta \right)\geq 0$$

In this case, $y^{*}\left(x,\theta \right)$ is defined as the follower-level function and is obtained by solving the follower problem.

$$\arg Min_{y\in Y}\;f\left(x,y,\theta \right)=y^{*}\left(x,\theta \right)$$

s.bg(x,y,θ)≥0

In this context, (x) represents the decision variable at the leader stage, and (y) represents the decision variable at the follower stage. (θ) is the sensitivity parameter, which may affect costs, capacities, or conditions. F(x,y,θ) and f(x,y,θ) are the objective functions at the leader and follower levels, respectively. G(x,y,θ) and g(x,y,θ) are the sets of constraints at the leader and follower levels, respectively.

**Fig. 2:**
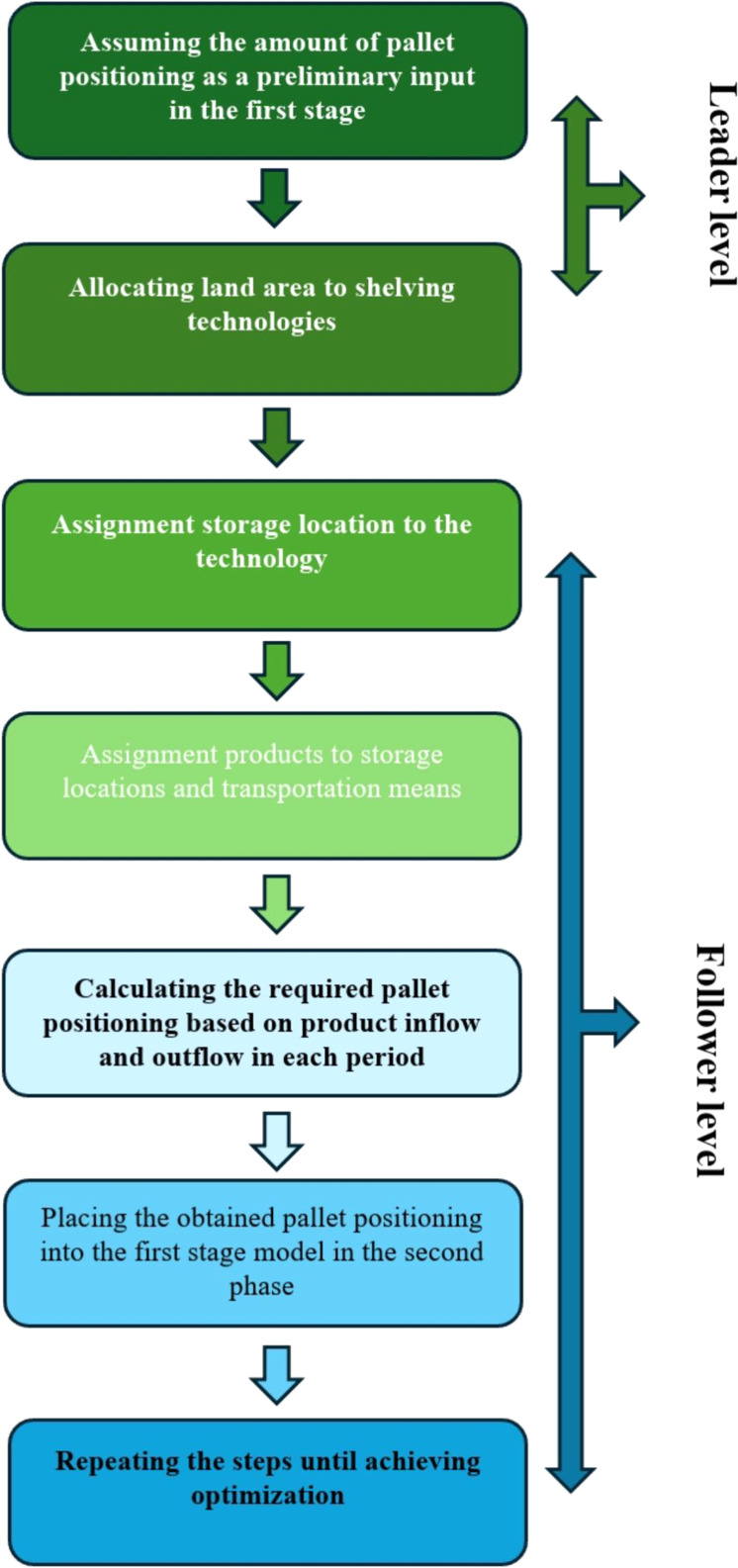
Stages of shelving technology selection and warehouse design.

In this study, the shelving technology selection problem is modelled as the leader-level decision within a bi-level optimization framework. At this level, the objective is to determine the optimal area allocation for each shelving technology, considering their respective construction costs and the required storage capacity. The decision variable at the leader level is the allocated area for each shelving technology, aiming to minimize construction costs while satisfying storage requirements. At the follower level, given the warehouse configuration defined by the leader model, storage locations are assigned to each selected shelving technology, followed by the assignment of products to those locations. Product allocation is constrained by weight limits and transportation vehicle capacities. The model is multi-periodic, where in each period, a probabilistic quantity of products enters the system for storage and a separate quantity is designated for retrieval. These fluctuations reflect demand uncertainty, which is modelled probabilistically. The maximum number of required shelf positions in each period is determined by the combination of incoming products and current inventory levels. This number serves as a dynamic input for the leader model. If the follower solution exceeds the available shelf capacity determined by the leader level, the model iterates by adjusting the area allocation and resolving the leader problem. This iterative process continues until convergence is achieved, ensuring that both capacity and cost objectives are satisfied. The overall procedure is conceptually depicted in Fig. 2.

**Fig. 3:**
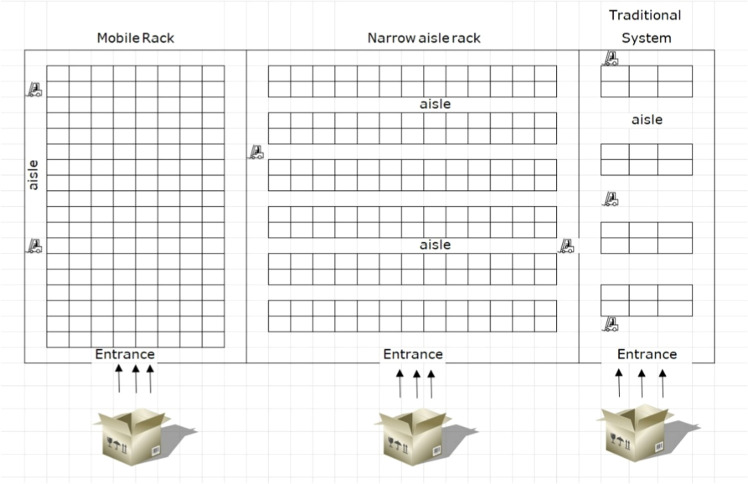
A view of the proposed design.

Initially, the available land is allocated among different shelving technologies based on the required number of storage positions. Following this allocation, shelving units are assigned to each selected technology, and subsequently, products are distributed across the shelves accordingly. During each planning period, a certain quantity of products enters the system for storage, while another portion is designated for order picking. Given the prevailing market uncertainties, the system is designed to maintain a certain level of inventory to ensure operational continuity and service reliability. The weight of each order is predetermined and must be compatible with the specifications and load-bearing capacities of the selected handling equipment. An illustrative representation of the proposed warehouse configuration and operational flow is provided in Fig. 3.

Next, the sets, parameters, and variables for the leader and follower models are presented.

**Leader Model Sets**

(v) Set of technologies

**Leader Model Parameters**

**Table:** 

$c_{v}$	Construction Cost of Technology
Ch	Excess Shelf Construction Cost. This value is constant and does not depend on the type of shelving technology employed.
Cb	Shelf Shortage Cost. This value is constant and does not depend on the type of shelving technology employed.
$f_{v}$	Efficiency of Each Technology
$b_{v}$	Layout Efficiency in Each Technology
$WTech_{v}$	Width of Each Shelf in Each Technology
$LTech_{v\;}$	Length of Each Shelf in Each Technology
W	Length of Land
L	Width of Land
Capacity	Required Shelf Quantity
$Nv_{j}$	Maximum Number of Shelves Allowed in Height in Each Technology

**Leader Model Variables**

**Table:** 

$a_{v}$	Area allocated to each technology
C	Excess shelf constructed
D	Shelf shortage constructed

**Leader Model Mathematical**

(1) $$\text{MinZ}=\sum_{v\in V}C_{v\;}*\;\frac{f_{v\;}*a_{v\;}}{WTech_{v}*LTech_{v\;}}+ch*C+cb*D$$

(2) $$\sum_{v\in V}a_{v\;}\leq W*\;L$$

(3) $$Capacity=\left(\sum_{v\in V}NV_{v\;}*\frac{f_{v\;}*a_{v\;}}{WTech_{v}*LTech_{v\;}}*b_{v}\right)-C+D$$

The objective function of the leader-level model seeks to minimize the total construction cost, which includes both the cost associated with each selected shelving technology and any penalties incurred for constructing more or fewer shelves than required. Constraint (2) ensures that the total area allocated to all shelving technologies does not exceed the available land area, thus respecting the spatial limitations of the warehouse. Constraint (3) serves as an equilibrium constraint, requiring that the aggregate number of positions allocated across all technologies matches the total number of positions required. This guarantees alignment between strategic-level design decisions and the actual storage capacity needed at the operational level.

**Follower Model Sets**

**Table:** 

I	Set of positions
O	Set of orders
R	Set of resources
T	Set of periods
K	Set of items

**Follower Model Parameters**

**Table:** 

$w_{o}$	Weight of each order
$e_{o}$	Area of each order
$cap_{r}$	Capacity of each resource
$c_{r}$	Cost of using each resource
$d_{ij}$	Travel cost from position i to position j
$h_{v}$	Cost of allocating each technology
N	the number of picks performed in each tour
N	the number of picks performed in each tour

**Follower Model Variables**

**Table:** 

$q_{iv}$	A binary variable that is 1 if position i is allocated to technology v, otherwise 0
$x_{ijt}$	A binary variable that is 1 if the picking path from position i to position j is in period t, otherwise 0
$y_{kit}$	A binary variable that is 1 if item k is allocated to position i in period t, otherwise 0
$m_{ort}$	A binary variable that is 1 if order o is picked by resource r in period t, otherwise 0
$u_{it}$	Auxiliary variable

(4) $$minz=\sum_{i}\sum_{j}\sum_{t}d_{ij}\;x_{ij}+\sum_{o}\sum_{r}\sum_{t}\;c_{r}\;m_{ort}\;$$

(5) $$\sum_{i}q_{iv}\;e_{i}\leq a_{v\;}\;\forall v$$

(6) $$\sum_{v}q_{iv}\leq 1\;\forall i$$

(7) $$\sum_{i}y_{kit}\leq M\sum_{i}q_{iv}\;\forall k,v,t$$

(8) $$\sum_{r}m_{ort}=1\;\forall o,t$$

(9) $$w_{o}\;m_{ort}\leq cap_{r}\;\forall o,r,t$$

(10) $$\sum_{i}y_{kit}=1\;\forall k,t$$

(11) $$\sum_{k}y_{kit}\leq 1\;\forall i,t$$

(12) $$\sum_{i}s_{kit}\geq st_{kt}\;\forall k,t$$

(13) $$\sum_{i}re_{kit}\geq ret_{kt}\;\forall k,t$$

(14) $$s_{kit}\leq M*y_{kit}\;\forall k,i,t$$

(15) $$re_{kit}\leq M*y_{kit}\;\forall k,i,t$$

(16) $$inv_{kit+1}=inv_{kit}+s_{kit}-re_{kit}\;\forall k,i,t$$

(17) $$\sum_{k}\left(inv_{kit}+s_{kit}\right)\leq Capacity\;\forall i,t$$

(18) $$\sum_{k}y_{kit}=\sum_{i}x_{ij}\;\forall i,j,t$$

(19) $$\sum_{k}y_{kit}=\sum_{j}x_{ij}\;\forall i,t$$

(20) $$u_{it}=n-1\;\forall i,t$$

(21) $$u_{it}-u_{jt}+nx_{ijt}=n-1\;\forall i,j,t\;i\neq j$$

(22) $$u_{jt}\geq 1\;\forall j,t$$

(23) $$u_{it}\geq 0\;\forall i,t$$

The objective function of the follower-level model aims to minimize the total cost of internal transportation, including both transportation distances and the operational costs associated with transport equipment. Constraint (5) ensures that the area utilized by each shelving technology does not exceed the amount of area allocated to it by the leader-level model. Constraint (6) guarantees that each storage position is assigned to exactly one shelving technology. Constraint (7) links the assignment of products to storage positions with the assignment of those positions to specific technologies, ensuring consistency across planning levels.

Constraint (8) governs the allocation of products to transportation vehicles, while Constraint (9) ensures that the capacity limitations of each vehicle are respected. Constraints (10) and (11) enforce exclusivity, stipulating that each product is assigned to exactly one position and each position is assigned to only one product, respectively. Constraints (12) and (13) define the quantities of products placed into and retrieved from the system during each planning period. Constraints (14) and (15) ensure that only products assigned to valid positions are eligible for placement or retrieval. Constraint (16) maintains inventory balance by computing the inventory level in each period as the difference between placements and retrievals. Constraint (17) restricts the inventory level and product placement quantity in each period to not exceed the total number of available positions. Constraints (18) and (19) define the relationship between storage location assignments and order picking operations, while Constraints (20) through (23) are used to eliminate sub-tours in the order picking route, preserving the feasibility of the solution with respect to routing.

Overall, the proposed model is an integrated, multi-period, bi-level framework that concurrently addresses strategic (shelving technology selection), tactical (storage location assignment), and operational (order picking) decisions, thereby enabling a comprehensive and coordinated approach to warehouse system optimization.

## Leader-follower game explanation

I In the proposed bi-level optimization model, the leader level initially assigns a provisional value to the required shelving capacity, denoted as the parameter Capacity. This parameter may be estimated based on market forecasts, expert insights, and the organization’s expansion strategies. At this stage, the available warehouse space is allocated among various shelving technologies, represented by the decision variable $a_{v}$, with the objective of minimizing the overall construction costs of the warehouse. The resulting allocation, $a_{v}$, serves as an input parameter for the follower-level model.

At the follower level, the number of storage positions assigned to each shelving technology is determined based on $a_{v}$, after which products are allocated to specific positions, and further assigned to the corresponding handling equipment. In each planning period, a certain quantity of products is placed into storage, while another quantity is retrieved to fulfill orders. To maintain service levels, a portion of products is held as inventory. These flows are modeled using the decision variables $s_{kit}$, $re_{kit}$, and $inv_{kit}$, representing stocked, retrieved, and inventory quantities of product *k* in period *t*, respectively.

**Fig. 4:**
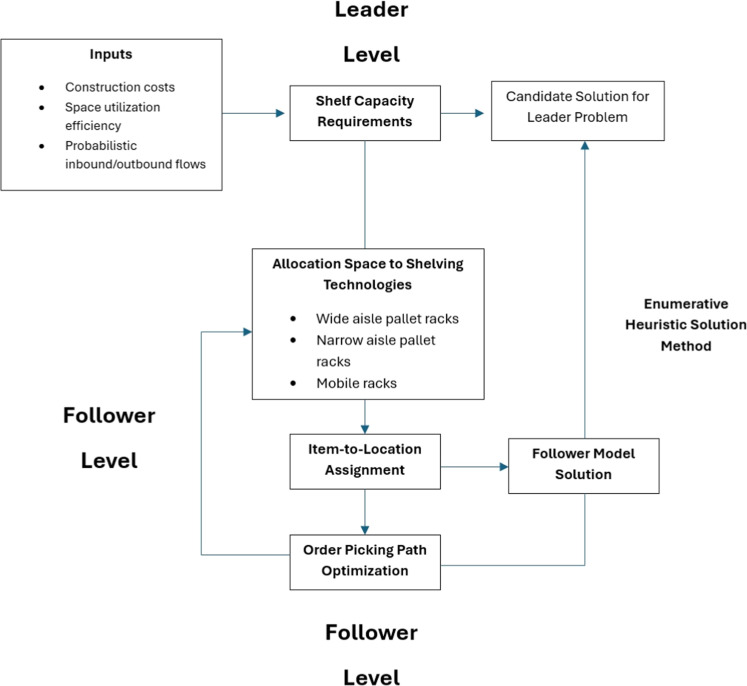
A view of the inclusion of a workflow diagram.

The maximum shelving capacity required at the follower level is derived from the maximum number of incoming products in any given period plus the inventory maintained across periods. Accordingly, at the follower level, Capacity becomes a decision variable rather than a parameter, and its value is determined endogenously by solving the model with the objective of minimizing transportation and equipment utilization costs. After solving the follower model, the updated value of Capacity is fed back to the leader level as a parameter. The leader model is then re-optimized based on this revised capacity requirement, generating a new allocation of $a_{v}$, which in turn updates the follower model. This iterative feedback loop continues until convergence is achieved and an optimal integrated solution is obtained for both hierarchical levels. In Fig. 4, the inclusion of a workflow diagram was presented.

After solving the model in the first stage, if the objective function does not improve, the model is re-executed. The process continues until no further improvement in the objective function is observed, at which point the model is considered to have converged and the optimal solution is obtained. The absence of improvement in the objective function thus serves as the convergence criterion for optimality.

## Modeling of probabilistic constraints

In optimization models involving uncertainty, variability in input parameters—such as demand, delivery times, costs, and environmental conditions—is explicitly accounted for. A variety of methods can be employed to manage such uncertainty, each with its own assumptions and applicability. Two widely used approaches are probabilistic modelling and robust optimization. In probabilistic models, uncertain parameters are represented as random variables with predefined probability distributions (e.g., normal, exponential, uniform). These models aim to optimize performance metrics by considering the expected behaviour of the system under uncertainty or by satisfying constraints with a certain confidence level. This approach is particularly useful when historical data or expert knowledge is available to accurately characterize the uncertainty. Robust optimization, by contrast, does not rely on specific probability distributions. Instead, it considers the worst-case scenarios within a defined uncertainty set, aiming to find solutions that remain feasible and near optimal under all plausible realizations of uncertain parameters. This technique is especially applicable in contexts where data is scarce, or risk aversion is critical. In the present study, the amount of product placement and retrieval in each planning period is modelled as a stochastic parameter. To account for this uncertainty, a probabilistic modelling approach is employed. By integrating demand fluctuations into the model, the proposed framework better reflects real-world dynamics and enhances the robustness and applicability of warehouse planning decisions under uncertainty.

The parametric form of the probabilistic constraints used in this study is presented below:

(24) $$Maxz=\sum_{i=1}^{n}C_{i}x_{i}$$

(25) s.t.Ax≤d

(26) $$P\left(\sum_{i=1}^{n}\tilde{a_{i}}x_{i}\leq \tilde{b}\right)\geq 1-\alpha$$

(27) $$x_{i}\geq 0,i=1,\ldots ,n$$

Where the constraint $P(\sum_{i=1}^{n}\tilde{a_{i}}x_{i}\leq \tilde{b})\geq 1-\alpha$ is a probability constraint.

In the presented model, only b ~ is random and has a normal distribution with mean $E(\tilde{b})$ and variance Var($\tilde{b})$. We want to rewrite the probability constraint $P(\sum_{i=1}^{n}\tilde{a_{i}}x_{i}\leq \tilde{b})\geq 1-\alpha$ as a normal constraint. Which is equivalent to:

(28) $$\sum_{i=1}^{n}a_{i}x_{i}\leq E\left(\tilde{b}\right)-Z_{1-\alpha }\sqrt{Var\left(\tilde{b}\right)}$$

In the presented model, the input and output parameters are probabilistic, all of which follow a normal distribution function with a specific mean and variance. As a result, in the proposed model, constraints 12 and 13 will be rewritten as probabilistic as follows:

(29) $$\sum_{i\in I}s_{kit}\;\geq E\left(st_{kt}\right)-z_{1-\alpha \;}\sqrt{var\left(st_{kt}\right)}\;\forall \;k\in K\;\forall \;t\in T\;$$

(30) $$\sum_{i\in I}\;re_{kit}\geq \;E\left(ret_{kt}\right)-z_{1-\alpha \;}\sqrt{var\left(ret_{kt}\right)}\;\forall \;k\in K\;\forall \;t\in T$$

In the following, the mathematical model will be solved using various methods, and a comparison between them will be presented. The probabilistic parameters used in the model, including the mean and standard deviation of demand as well as inbound and outbound flows, were derived from five years of historical data recorded in the company’s warehouse management system. These statistical estimates were subsequently validated and, where applicable, adjusted through consultations with experienced warehouse planners to ensure alignment with current operational conditions.

## Method validation

Bi-level optimization is a class of hierarchical optimization problems characterized by two interconnected levels of decision-making: a leader level, where strategic decisions are made, and a follower level, which responds optimally to the decisions imposed by the leader. The complexity of such problems arises from the dependency between the two levels, necessitating advanced computational methods for effective solutions.

One efficient approach to address bi-level problems is the enumerative heuristic algorithm. In this method, the leader’s decision space is discretized, and all feasible combinations of the leader-level decision variables are systematically evaluated. For each combination, the corresponding follower-level subproblem is solved. This procedure ensures thorough exploration of the solution space and yields a near-optimal solution for the entire hierarchical structure. The implementation of this method involves several key steps. First, the decision space for the leader-level variables is constructed, generating a finite set of candidate solutions. For each candidate, the follower-level problem is solved independently, yielding the optimal response to that specific leader decision. The outputs from the follower level are then fed back into the leader level, allowing for evaluation of the overall objective function. This iterative process continues until convergence to the best-performing solution is achieved.

In this study, the proposed bi-level problem is solved deterministically using the General Algebraic Modelling System (GAMS) software. The model considers three shelving technologies commonly employed in warehouse design:

Wide-aisle pallet racks (traditional layout), Narrow-aisle pallet racks, Mobile racks

Each of these technologies requires specific types of equipment and offers different levels of space utilization efficiency. The selection of shelving technology involves trade-offs among setup costs, equipment requirements, and internal transportation costs. Additionally, there may be discrepancies between the actual and desired number of shelving positions, resulting in either excess or deficiency. The model accurately quantifies the construction costs for each technology and the penalties associated with over- or under-provisioning, thereby supporting optimal design and planning decisions.

## Solution of the proposed model

To evaluate the performance of the proposed model, a numerical example was developed and solved. In this case study, the construction of a warehouse is considered, with the objective of selecting the most appropriate shelving technology from among three available alternatives over a five-period planning horizon. The warehouse operation involves five types of handling equipment and five types of products.

Initially, a required storage capacity of 10,000 pallet positions is assumed as the input for the leader model. Based on this assumption, the leader model suggests allocating 2278 pallet positions using the first shelving technology and 520 pallet positions using the second technology. This initial configuration is then provided as input to the follower model.

In the leader problem, the total warehouse floor area was discretized into intervals of 500 m² for each shelving technology under consideration. All combinations of these discretized allocations that satisfied the total area constraint were generated, resulting in an initial pool of 144 candidate configurations. A pruning step was then applied to remove any configuration whose total storage capacity fell below the minimum demand requirement derived from the stochastic input data. For each remaining configuration, the follower problem was solved using the GAMS implementation, and the resulting objective function value was fed back into the leader problem. The enumeration continued until all feasible configurations were evaluated. This procedure ensures that the search space is exhaustively explored while avoiding the computational overhead of evaluating infeasible or clearly suboptimal designs.

In the follower stage, the input and output rates for each period are modelled as normally distributed random variables to capture demand uncertainty. The follower model computes the actual required number of pallet positions based on product placements, retrievals, and inventory levels. In this instance, the result shows a total requirement of 22,967 pallet positions, which significantly exceeds the initial capacity assumed in the leader stage.

Given this discrepancy, the leader model is re-solved using the updated capacity requirement. In the second iteration, the model recommends allocating 817 pallet positions to the first technology and 1980 pallet positions to the second technology. The follower model is then solved once again using this revised configuration.

Since no significant changes are observed in the demand patterns—as reflected by the standard deviation of the input/output rates across the five periods, the newly suggested layout from the leader model is accepted as final. Thus, the problem is considered solved, and the iterative process successfully converges with an optimal warehouse design that satisfies both strategic and operational requirements under probabilistic conditions.

## Sensitivity analysis of the leader model

**Chart 1:**
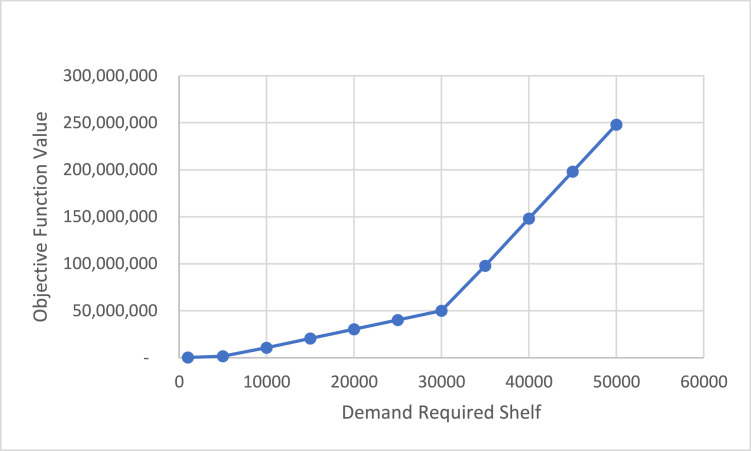
Changes in the objective function for increasing the demand for the desired shelf.

In Chart 1 illustrates the variation in the objective function value as the required number of pallet positions increases. In the initial stages, where storage demand is relatively low, the model selects only the wide-aisle pallet rack technology due to its lower setup cost and suitability for general-purpose use. However, as the required number of shelves increases, the model gradually shifts toward the use of narrow-aisle pallet rack technology, which offers higher space utilization efficiency despite its higher initial investment cost. The allocation pattern and utilization of each shelving technology across different capacity levels are summarized in Table 3.

**Table 3:** The rate of use of the assigned technologies.

Demand Required Shelf	Assigned Technology			Objective Function Value
	1	2	3	
1000	525	0	0	315,040
5000	2625	0	0	1575,200
10,000	2278	2	520	10,743,000
15,000	1715	0	1084	20,541,000
20,000	1184	2	1647	30,351,000
25,000	589	1	2210	40,141,000
30,000	22	3	2773	49,951,000
35,000	0	0	2800	98,000,000
40,000	0	0	2800	148,000,000
45,000	0	0	2800	198,000,000
50,000	0	0	2800	248,000,000

Given the inherent limitations of shelving technologies, dimensional modifications are not practically feasible. Therefore, dimensional analysis has been excluded from the current stage of the study.

## Sensitivity analysis of the follower model

In the follower model, an integrated formulation of the item location assignment and order picking problems is presented. To evaluate the model, an illustrative example is initially solved using the data provided in Table 4.

**Table 4:** An example of the integrated model for item location assignment and order picking.

Number of Positions	Number of Products	Number of Equipment	Number of Periods	Number of Technologies
10	5	5	5	3

Given the explanations regarding the dimensional characteristics, the number of shelving technologies, and the diversity of equipment, dimensional analysis was deemed impractical and therefore not conducted. Although the required shelf capacity should ideally be derived from the leader model, in this case, to isolate and evaluate the follower model, a default capacity value was assumed. Accordingly, the area allocated to each shelving technology was set at 3000 square meters. For the purposes of the sensitivity analysis, an allocation of 3000 m² per shelving technology was assumed, corresponding to a conventional warehouse plot measuring 60 m in length and 50 m in width, which is a typical configuration for warehouse construction in practice.

**Chart 2:**
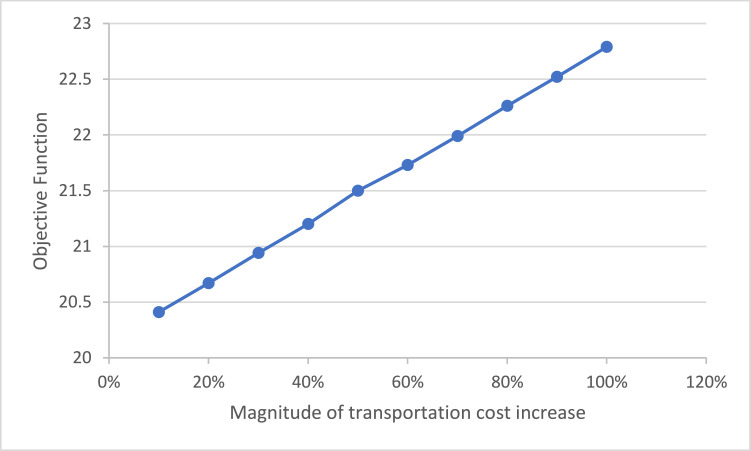
Changes in the objective function as transportation costs increase.

To assess the sensitivity of the follower model to changes in transportation costs, a scenario-based analysis was performed. The results indicate that a 10 % increase in transportation costs leads to an approximate 0.2 % increase in the objective function value. As illustrated in Chart 2, this relationship exhibits a linear trend, whereby increasing transportation costs proportionally elevate the total objective value.

From a managerial perspective, the results of the sensitivity analysis offer practical guidance for warehouse planners in aligning investment decisions with operational performance objectives. For example, increasing the allocation of floor space to a particular shelving technology may substantially reduce transportation costs up to a certain threshold, beyond which further expansion yields minimal additional benefit. Similarly, under conditions of high demand variability, technologies with greater flexibility in storage capacity may provide more consistent cost savings and space utilization. By identifying such thresholds and performance trade-offs, planners can prioritize resource allocation, select appropriate technologies, and avoid overinvestment in capacity that does not materially improve overall performance.

## Sensitivity analysis of the integrated model considering probabilistic conditions

In the proposed integrated model, the rates of inbound and outbound item flows are considered probabilistic, with the mean and variance of these rates defined for each planning period. The model is solved under the operational conditions specified in Table 4, and multiple scenarios are examined by varying the initial estimates for the required number of storage positions. The corresponding results are reported in Table 5.

**Table 5:** Sensitivity analysis results of the integrated model.

ZL(2)+ZF(2)	ZL(2)	ZF(1)	$a_{v\;}\left(2\right)$			Capacity (2)	ZL (1)	$a_{v\;}\left(1\right)$			Capacity (1)
			3	2	1			3	2	1	
32,140,229	32,140,000	229	1750	0	1050	20,917	315,040	0	0	525	1000
34,980,243	34,980,000	243	1913	2	883	22,359	1575,200	0	0	2625	5000
36,154,249	36,154,000	249	1980	3	817	22,967	10,743,000	520	2	2278	10,000
31,863,227	31,863,000	227	1733	4	1061	20,774	20,541,000	1084	0	1715	15,000
32,887,233	32,887,000	233	1793	0	1005	21,293	248,000,000	2800	0	0	50,000

As shown in Table 5, an increase in the initially estimated number of required storage positions leads to a corresponding rise in the implementation and construction costs of the selected shelving technologies. Since the actual number of required positions is influenced by probabilistic input/output rates and inventory levels, the application of a Bi level modelling approach effectively reduces overall costs. Initially, the storage capacity is determined based on expert judgment and entered into the model. The subsequent solution process of the model relies on actual inbound and outbound flow data, which are independent of the expert-provided capacity estimates. Consequently, when experts suggest a higher capacity, the actual inbound and outbound quantities remain unchanged. The model, therefore, performs its calculations based on the real observed flows rather than the expert-assumed capacity. This explains why, in some cases, an increase in the expert-estimated capacity can result in a reduction of the objective function value, as the model optimizes according to the true operational data rather than the nominal capacity provided by experts. This approach facilitates a more accurate and practical determination of shelving technology needs, ensuring alignment between system design and operational realities.

## Time complexity of the proposed model

In optimization problems—particularly in combinatorial and hierarchical models commonly applied in real-world scenarios—time complexity is a critical metric for evaluating the efficiency of algorithms and mathematical formulations. Time complexity refers to how the computational time required to solve a model scale with the size of the problem, such as the number of decision variables, constraints, or input parameters. This concept provides an analytical foundation for assessing the scalability and computational robustness of a model as data volume increases [18,19].

The relevance of time complexity becomes even more pronounced in bi-level optimization models, where solving the upper-level problem necessitates repeated resolution of the lower-level problem for every configuration of leader-level decisions [20]. In general, time complexity analysis involves developing an estimation function that links the dimensions of the model to the required CPU processing time. Within this framework, problem dimensions—such as the number of constraints and variables—are treated as independent variables, while CPU time serves as the dependent variable. Computational effort in mathematical programming models is typically influenced by the number of constraints and decision variables. For instance, if the average processing time per constraint is 0.01 s, the overall solution time can be estimated accordingly. A similar relationship applies to the number of decision variables. This analytical approach enables researchers to project computational requirements and assess the tractability of their models under various scenarios.

**Chart 3:**
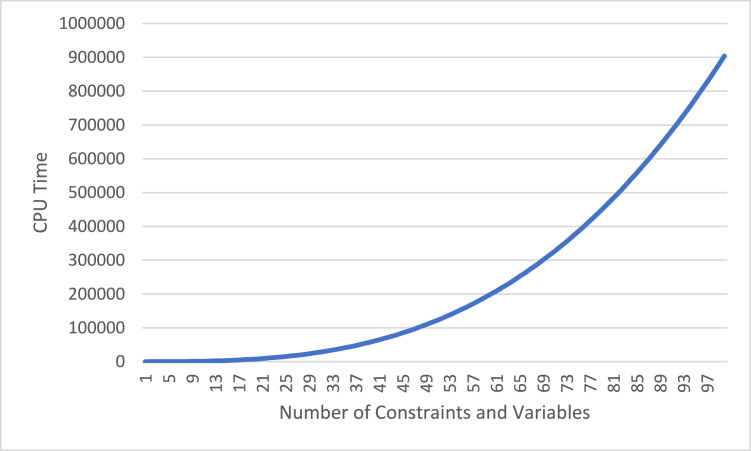
The effect of Number of Decision Variables and Constraints on CPU Time.

In the proposed model, based on the structure of the constraints, the number of constraints is given by the equation: f(v+i+kvt+ot+ort+4kt+7it+3kit+iot+okt+2), And considering the variables of the proposed model, the equation f(v+2+iv+ijt+kit+ort+okt) represents the number of variables. Assume that there are 3 shelving technologies, 5 locations, 3 orders, 3 products, 3 vehicles, and 5 time periods. In this case, there are 423 constraints and 189 variables. This type of computation has been performed for various values of variables and constraints, and the results are presented in Chart 3.

In general, due to the high initial construction costs associated with advanced shelving technologies such as mobile racks and narrow-aisle pallet racks, the model tends to favor traditional wide-aisle pallet racks when the demand for shelving capacity is relatively low. However, when a substantial number of shelving units is required and space availability is constrained, the model shifts preference toward technologies that offer higher space utilization efficiency. Notably, certain technologies incur significantly higher transportation costs; thus, a trade-off must be established between transportation expenses and shelving construction costs. In the proposed model, this balance is effectively achieved by simultaneously accounting for all relevant factors, including spatial constraints, equipment requirements, and operational costs.

Overall, the proposed framework serves as a practical decision-support tool for warehouse operations managers. By enabling comprehensive sensitivity analyses, the model facilitates informed decision-making in the early stages of warehouse construction and shelving technology selection, ultimately supporting the achievement of cost-effective and efficient warehouse system design.

## Limitations

One of the key limitations of the proposed integrated model is its computational complexity. Due to the NP-hard nature of simultaneously solving warehouse design, storage location assignment, and order picking—particularly within a bi-level optimization framework—exact solution methods become impractical for medium- and large-scale problem instances. To overcome this limitation, the use of metaheuristic algorithms offers a promising approach, enabling the generation of high-quality solutions within reasonable computational time. In this study, only demand values as well as inbound and outbound flows are treated as stochastic parameters; in other words, only the right-hand-side terms of the model are subject to uncertainty. This constitutes a limitation of the present work. Future studies may extend the approach by incorporating additional parameters, such as operational costs and other relevant factors, as stochastic variables. In this study, the demand is assumed to follow a normal distribution, which was selected as a simplifying assumption consistent with the statistical characteristics of the historical demand data used. However, we recognize that this assumption may not always be valid in real-world contexts, and the model could be extended to accommodate alternative distributions or to assess its robustness under distributional shifts. Future research could develop and analyze a sequential baseline model, in which strategic, tactical, and operational decisions are addressed separately, to quantitatively demonstrate the performance gap identified in the literature between integrated and non-integrated approaches. The proposed model could be extended in several directions. First, its application to larger warehouses with more complex layouts would allow for evaluating scalability, computational efficiency, and potential operational trade-offs that may not appear in smaller-scale configurations. Second, incorporating real-time adaptability—through rolling-horizon or event-triggered re-optimization—would enable the framework to respond effectively to dynamic changes in demand, inventory, or operational conditions, thereby maintaining solution quality and decision-making relevance. Third, integrating labor scheduling (including shift planning, skill allocation, and overtime policies) and robotics (such AS/RS, AGVs, and AMRs) into the optimization framework would provide a more comprehensive decision-support tool, jointly addressing strategic design, tactical storage assignment, operational routing, and human/robot resource utilization. These extensions would significantly enhance the practical applicability of the model in modern, dynamic, and technology-driven warehouse environments.

## Ethics statements

This work did not involve human subjects, animal experiments, or data collected from social media platforms.

## CRediT authorship contribution statement

**Mohammadreza Farhadi Moghadam:** Conceptualization, Methodology, Software, Formal analysis, Writing – original draft. **Kaveh Khalili damghani:** Validation, Resources, Data curation, Writing – review & editing. **Vahidreza Ghezavati:** Supervision, Methodology, Writing – review & editing. **Alireza Rashidi Komijan:** Validation, Supervision, Writing – review & editing.

## Declaration of competing interest

The authors declare that they have no known competing financial interests or personal relationships that could have appeared to influence the work reported in this paper.

## Figures and Tables

**Table:** 

v	Set of technologies

## Data Availability

Data will be made available on request.

## Appendix 1

**Notation glossary summarizing**

**Leader Model set**

**Follower Model Sets** i: Set of positions o: Set of orders r: Set of resources t: Set of periods k: Set of items

**Leader Model Parameters**

$c_{v}$: Construction Cost of Technology

Ch: Excess Shelf Construction Cost. This value is constant and does not depend on the type of shelving technology employed.

Cb: Shelf Shortage Cost. This value is constant and does not depend on the type of shelving technology employed.

$f_{v}$: Efficiency of Each Technology

$b_{v}$: Layout Efficiency in Each Technology

$WTech_{v}$: : Width of Each Shelf in Each Technology

$LTech_{v\;}$: : Length of Each Shelf in Each Technology

W: Length of Land

L: Width of Land

Capacity: : Required Shelf Quantity

$Nv_{j}$: Maximum Number of Shelves Allowed in Height in Each Technology

**Follower Model Parameters**

$w_{o}$: Weight of each order

$e_{o}$: Area of each order

$cap_{r}$: : Capacity of each resource

$c_{r}$: Cost of using each resource

$d_{ij}$: Travel cost from position i to position j

$h_{v}$: Cost of allocating each technology

N: the number of picks performed in each tour

**Leader Model Variables**

$a_{v}$: Area allocated to each technology

C: Excess shelf constructed

D: Shelf shortage constructed

**Follower Model Variables**

$q_{iv}$: A binary variable that is 1 if position i is allocated to technology v, otherwise 0

$x_{ijt}$: A binary variable that is 1 if the picking path from position i to position j is in period t, otherwise 0

$y_{kit}$: A binary variable that is 1 if item k is allocated to position i in period t, otherwise 0

$m_{ort}$: A binary variable that is 1 if order o is picked by resource r in period t, otherwise 0

$u_{it}$: Auxiliary variable

**Bi level Notation**

X: variable at the leader stage

Y: variable at the follower stage

θ: sensitivity parameters

F(x,y, θ), f(x,y, θ): objective functions at the leader and follower level

G(x,y, θ), g(x,y, θ): set of constraints at the leader and follower level

## Appendix 2

**Follower Model Code**

**sets** i Locations /1 × 10/ o Orders/1 × 5/ r resources /1 × 5/ t Periods /1 × 5/ v technologies /1 × 3/ alias(o,k) alias(o,l) alias(i,j); scalar n /5/ b big number /1000,000/ cdemand /0.01/; parameters d(i,j),w(o),cap(r),c(r),f(v),e(i),h(v),st(k,t),ret(k,t),hc(k)

$onecho>text2.txt par=*d* rng=*d*!a1 rdim=1 cdim=1 par=*w* rng=*w*!a1 rdim=1 par=cap rng=cap!a1 rdim=1 par=*c* rng=*c*!a1 rdim=1 par=*f* rng=*f*!a1 rdim=1 par=*e* rng=*e*!a1 rdim=1 par=*h* rng=*h*!a1 rdim=1 par=st rng=st!a1 rdim=1 cdim=1 par=ret rng=ret!a1 rdim=1 cdim=1 par=hc rng=hc!a1 rdim=1

$offecho

$ call gdxxrw test2.xlsx @text2.txt

$ gdxin test2.gdx

$ load d w cap c f e h st ret hc

$ gdxin variables z; binary variable x(i,j,t) y(k,i,t) m(o,r,t) q(i,v); positive variable u(i,t) s(k,i,t) re(k,i,t) inv(k,i,t) demand; u.up(i,t)=1000,000; s.up(k,i,t)=1000,000; re.up(k,i,t)=1000,000; inv.up(k,i,t)=1000,000; demand.up=1000,000; equations obj c1 c2 c3 c4 c5 c6 c7 c8 c9 c10 c11 c12 c13 c14 c15 c16 c17 c18; obj..*z* = *e*=sum((i,j,t),d(i,j)*x(i,j,t))+sum((o,r,t),c(r)*m(o,r,t))+demand*cdemand; c1(v)..sum(i,e(i)*q(i,v))=*l* = *f*(v); c12(i)..sum(v,q(i,v))=*l* = 3; c2(i,t).. sum(k,y(k,i,t))=*l* = *b**sum(v,q(i,v)); c3(o,t)..sum(r,m(o,r,t))=*g* = 1; c4(r,t)..sum(o,w(o)*m(o,r,t))=*l*=cap(r); c5(k,t)..sum(i,y(k,i,t))=*e* = 1; c6(i,t)..sum(k,y(k,i,t))=*l* = 1; c7(i,t)..sum(k,y(k,i,t))=*e*=sum(j,x(i,j,t)); c8(j,t)..sum(k,y(k,j,t))=*e*=sum(i,x(i,j,t)); c9(i,j,t)$(ord(i)<>ord(j)and ord(i)>=2 and ord(j)>=2)..u(i,t)-u(j,t)+*n**x(i,j,t)=*l* = *n*-1; c10(i,t)..u(i,t)=*l* = *n*-1; c11(j,t)..u(j,t)=*g* = 0; c13(k,t)..sum(i,s(k,i,t))=*g*=st(k,t); c14(k,t)..sum(i,re(k,i,t))=*g*=ret(k,t); c15(k,i,t)..s(k,i,t)=*l* = *b**y(k,i,t); c16(k,i,t)..re(k,i,t)=*l* = *b**y(k,i,t); c17(k,i,t)..inv(k,i,*t* + 1)=*e*=inv(k,i,t)+*s*(k,i,t)-re(k,i,t); c18(i,t)..sum(k,inv(k,i,t)+*s*(k,i,t))=*l*=demand; x.fx(i,i,t)=0; u.up(i,t)=*n*-1; u.lo(i,t)=1; model stage2/all/; option limcol=100,000,limrow=100,000; option reslim=360,000; option optca=0, optcr=0; option iterlim=10,000,000; solve stage2 using mip min z;
